# Optimizing the Color Shapes Task for Ambulatory Assessment and Drift Diffusion Modeling: A Factorial Experiment

**DOI:** 10.2196/66300

**Published:** 2025-10-01

**Authors:** Sharon Haeun Kim, Jonathan G Hakun, Yanling Li, Karra D Harrington, Daniel B Elbich, Martin J Sliwinski, Joachim Vandekerckhove, Zita Oravecz

**Affiliations:** 1 Department of Human Development and Family Studies The Pennsylvania State University University Park, PA United States; 2 Department of Social Data Analytics Pennsylvania State University University Park United States; 3 Department of Neurology College of Medicine The Pennsylvania State University Hershey, PA United States; 4 Department of Psychology The Pennslyvania State University University Park, PA United States; 5 Department of Public Health Services College of Medicine The Pennsylvania State University Hershey, PA United States; 6 Center for Healthy Aging The Pennsylvania State University University Park, PA United States; 7 Department of Cognitive Sciences University of California, Irvine Irvine, CA United States; 8 Institute for Computational and Data Sciences Pennsylvania State University University Park United States

**Keywords:** subtle cognitive decline, computational cognitive markers, smartphone-based cognitive testing, drift diffusion model, Bayesian multilevel modeling, mobile phone

## Abstract

**Background:**

Recent advances in cognitive digital assessment methodology, including high-frequency, ambulatory assessments, promise to improve the detection of subtle cognitive changes. Computational modeling approaches may further improve the sensitivity of digital cognitive assessments to detect subtle cognitive changes by capturing features that map onto core cognitive processes.

**Objective:**

We explored the validity of a brief smartphone-based adaptation of a visual working memory task that has shown sensitivity for detecting preclinical Alzheimer disease risk. We aimed to optimize properties of the task for computational cognitive feature extraction with drift diffusion modeling.

**Methods:**

We analyzed data from 68 participants (n=47, 69% women; n=55, 81% White; mean age 49, SD 14; range 24-80 years) who completed 60 trials for each of 16 variations of a visual working memory binding task (the Color Shapes task) on smartphones, over an 8-day period. A drift diffusion model was fit to the response time and accuracy data from the task. We experimentally manipulated 3 properties of the Color Shapes task (study time, probability of change, and choice urgency) to test how they yielded differences in key drift diffusion model parameters (drift rate, initial bias toward a response option, and caution in decision-making). We also evaluated how an additional task property, the test array size, impacted responses across all conditions. For array size, we tested a *whole display* of 3 shapes against a *single probe* of 1 shape only.

**Results:**

The 3 task property manipulations yielded the following results: (1) increasing the ratio of *different* responses was credibly associated with higher initial bias toward the *different* response (mean 0.06, SD 0.02 for the whole display; mean 0.15, SD 0.02, for the single probe condition); (2) increasing the choice urgency during the test phase was credibly associated with decreased caution in decision-making in the single probe condition (mean −0.04, SD 0.02) but not in the whole display (mean −0.01, SD 0.02); and (3) contrary to expectation, longer study times did not yield a credibly faster drift rate but produced credibly slower ones for the whole display condition (mean −0.28, SD 0.05) and a null effect for the single probe condition (mean 0.01, SD 0.05). In addition, as expected, we found that individual differences in drift rate were associated with age in both array sizes (*r*=−0.45 with Bayes factor=191), with older participants having a slower drift rate. Older participants also showed higher caution (*r*=0.42 with Bayes factor=80.76) in the single probe condition.

**Conclusions:**

We identified a version of the Color Shapes task optimized for smartphone-based cognitive assessments in real-world settings, with data designed for analysis through computational cognitive modeling. Our proposed approach can advance the development of tools for efficient and effective early detection and monitoring of risk for Alzheimer disease.

## Introduction

### Background

Conventional cognitive testing is widely used to diagnose neurocognitive impairments from various clinical conditions. However, many of these conditions have a long preclinical phase, characterized by cognitive changes that are more subtle than what most conventional cognitive tests are designed to detect [1,2]. In other words, while cognitive performance summary scores (typically obtained from 1-time assessment) may be sufficient for detecting overt impairments in neurodegenerative diseases, they often lack the sensitivity to capture the early-stage changes in subtle cognitive subprocesses. Ratcliff and McKoon [3] have suggested that cognitive and neuropsychological testing could capitalize on advances in cognitive modeling to move beyond static performance summary scores and better assess the latent cognitive dynamics. With advances in digital health tools, smartphone-based cognitive assessments have shown promise for capturing subtle processes in cognitive performance daily contexts (ie, ambulatory assessment), facilitating more representative and richer measurements [4,5]. Here, we introduce a smartphone-based implementation of a working memory task that evaluates a cognitive function that has been linked with early-stage Alzheimer disease (AD) risk [6,7].

Recent evidence suggests that sensitive measures of subtle cognitive processes may be captured through repeated ambulatory cognitive assessments with smartphones [8], particularly when combined with computational cognitive modeling [9]. High-frequency ecological momentary assessments [10] of cognitive functioning across days produces a rich data stream across time and daily life contexts [11], providing a representative sample of daily cognition. These repeated assessments in natural contexts also mitigate reliability concerns [8], while preserving ecological validity [12], and permit the implementation of computational methods to effectively explore subtle within-person cognitive processes [13]. However, many cognitive tasks are designed for laboratory administration, and the resulting performance scores are often summarized via simple summary statistics. Here, we show how subtle cognitive processes underlying ambulatory reaction time and accuracy data can be captured with cognitive modeling. Our approach emphasizes the advantages of identifying individual differences in cognitive features via state-of-the-art computational cognitive psychometric modeling from repeated ambulatory assessments of cognitive performance.

A consistent theme in the cognitive modeling literature highlights the benefits associated with cognitive model–based approaches, which support the widely used scaling transformations [14,15], assist in identifying potential confounds [6,16], and improve parameter estimation stability [17]. When it comes to evaluating processing speed for example, cognitive modeling can jointly consider both response time (RT) and choice behavior and contribute to greater analytical sensitivity than mean-level insights alone [3]. Cognitive psychometric models [18] provide mathematically disciplined approaches for simulating data-generating mechanisms, distilling observed behavioral scores, and extracting individual differences in their latent cognitive features. In summary, this approach can identify subtle latent processes underlying manifest cognitive performance data and explore individual differences therein to capture latent mechanisms and dynamic processes [19].

There has been a growing shift toward adopting cognitive models to diagnostic challenges posed by early AD, such as a need to distinguish AD-specific markers from other neuropsychological conditions, normative age-related declines, and practice effects [3,7,16]. Cognitive modeling approaches have shown promise in identifying early cognitive markers of dementia risk, such as reduced episodic memory [20], higher task-switching costs [21], declines in attentional control [22], and higher performance inconsistency [23]. Moreover, cognitive modeling can parse out individual differences in these cognitive features linked to clinically relevant person-level characteristics, such as age [24], genetic and familial risks [6,20], and cognitive impairment status [3,9]. The drift diffusion model (DDM) framework has also been increasingly applied to characterize latent cognitive processes underlying neurological conditions [25-28] and age-related cognitive changes [29,30].

Parra et al [6] identified visual working memory binding as one of the earliest underlying functions impacted in preclinical AD, particularly deficits in “binding” visual features. This feature-binding deficit has demonstrated sensitivity and specificity across a range of at-risk populations, validated as a reliable early cognitive marker in symptomatic and asymptomatic mutation carriers [6,25], individuals with mild cognitive impairment and AD [26,30], and individuals across the clinical continuum with elevated AD-related biomarkers [27,31]. The original task was designed to isolate visual feature binding by using novel, abstract shapes and randomized color-shape pairings [6]. The abstract shapes were selected to make it difficult to attach a label to them (eg, triangle and square), thus preventing undesirable verbal encoding instead of relying on visual working memory. Building on this paradigm, we tested a mobile adaptation of this task, which we will refer to as the “Color Shapes” task [5]. Previous implementations of the Color Shapes task for mobile administration have been largely web based [28,29] and focused on analyzing aggregated mean performance data across all administrations and did not explore the cognitive processes through computational modeling.

To address this gap, we propose that cognitive modeling of repeated administrations of Color Shapes task can further enhance our ability to capture subtle cognitive change processes. This approach can formally assess the validity of the model by evaluating fit with observed data [19]. One such model, the DDM [32], characterizes decision-making as a noisy process of evidence accumulation toward response options and can disentangle into hypothesized generative cognitive processes. The DDM captures the latent processes underlying observed performance of RT and accuracy [3,14,33]. By fitting the DDM to RT and accuracy data, we can disentangle subtle underlying cognitive processes, such as rate of evidence accumulation, caution in decision-making, and response biases [32,34]. For instance, individual differences in the speed of evidence accumulation and caution can elucidate important idiosyncrasies in decision-making so that we can better identify those at risk for AD [20,35].

### Objectives

The aim of this study was twofold: (1) to conduct an experimental validation of the DDM analytic approach in the context of RT and accuracy data from the Color Shapes task and (2) to identify an ambulatory Color Shapes task version that is most optimal for DDM-based data analysis. We propose that the DDM-based computational modeling approach can extract useful signals that can serve as novel digital cognitive markers of risk for subtle cognitive decline. Consistent with the literature on cognitive testing and aging, we hypothesized the following effects on cognitive features in response to our experimental manipulations: (1) allowing for longer study time would increase the evidence accumulation rate, (2) showing trials with a higher number of *different* trials (48/60, 80% *different* trials) would bias the starting point of the decision process toward *different*, and (3) imposing higher decision urgency (3000 ms limit) would lower the decision boundary, corresponding to lower response cautiousness. Overall, our study aimed to lay the groundwork for this approach and illustrate the corresponding statistical tools for data analysis. As a corollary to our optimization effort, we also considered which test array size produces data in ambulatory setting that are in line with the assumptions of the DDM (ie, single shot decision-making) and measuring visual working memory (as opposed to memory relying on verbal encoding). For this, we varied 2 test phase formats: simultaneous presentation of 3 visual elements phase (labeled as *whole display*) versus simply showing a single item in the test phase (*single probe*).

## Methods

### Ethical Considerations

This study was approved by the Pennsylvania State University Institutional Review Board (STUDY00018787). All procedures were conducted in accordance with the ethical principles outlined in the Declaration of Helsinki and all applicable guidelines and regulatory standards. All participants provided written informed consent before participation in the study. All data were fully deidentified before analysis and handled in anonymized form. Participants received compensation of up to US $100 for completing the study.

### Participant Recruitment

We recruited 69 adults from Pennsylvania (n=47, 69% women; n=21, 31% men) on the web via ResearchMatch, a nonprofit, National Institutes of Health–funded national registry established through the Clinical Translational Science Award program to support medical study participation [36,37]. Participants were screened via telephone to assess eligibility. Participant inclusion criteria are presented in Textbox 1.

**Participant inclusion criteria.**

- Aged >18 years
- Fluent in English
- Has access to a reliable internet connection
- Does not have a motor or visual impairment that would interfere with operating a smartphone
- Does not have a history of neurological injury or disease (eg, stroke and seizures)

After an initial remote onboarding session (conducted via videoconference), participants were asked to complete cognitive tasks (Color Shapes trials; details in the measures section) for 8 days for approximately 20 minutes each day. More information is provided in the design and procedure section, which follows the brief description of the task and the computational modeling framework chosen to analyze the data.

### Measures

#### Demographics

Participants responded to gender, age, race and ethnicity, ethnic status, and level of education. Participants were asked whether they had ever tested positive for COVID-19 (data were not analyzed). Basic health information was provided on ResearchMatch upon sign-up (eg, BMI, medical conditions, and medication).

#### The Color Shapes Task

The Color Shapes task is a visual array change detection task where participants were asked to determine whether the combination of features (color and shape) among visual objects distributed throughout the array change between study and test arrays. In our study, participants were asked to study a set of 3 abstract shapes, each with a unique color. After a brief 900-ms delay, they were presented with a test array and asked to determine if the stimuli contained in the test array contained the same combination of shape and color as the study array. To identify an optimal combination of task parameters for mobile administration and drift diffusion modeling, we experimentally manipulated 4 features of the task in a within-persons 2 (study duration)×2 (probability of change)×2 (response duration)×2 (probe type) full factorial experimental design. Specifically, each trial of the Color Shapes task began with a fixation cross displayed for 500 ms after which 3 colored abstract shapes appeared for either a short (500 ms) or longer (2000 ms) period (study duration). Participants were asked to study the specific combinations of shape and color presented in the study array objects.

**Figure 1:**
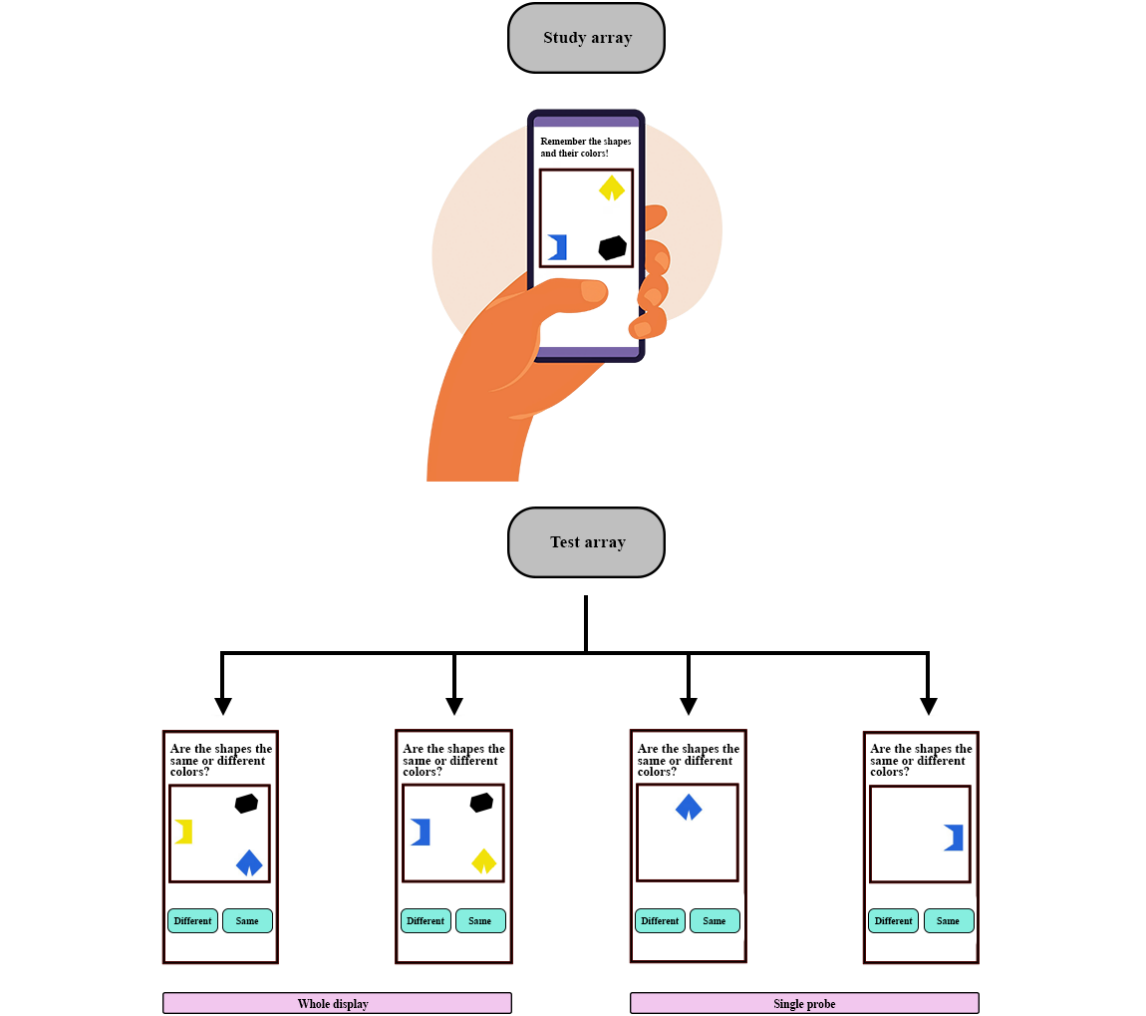
Study and test arrays of the Color Shapes task. An illustration of the Color Shapes task showing the study array and test array with four configurations from left to right: (1) different trial with whole display (3-colored shapes) shown in the test array, (2) same trial with whole display (3-colored shapes) shown in the test array, (3) different trial with single probe (1-colored shape) shown in the test array, and (4) same trial with single probe (1-colored shape) shown in the test array.

The abstract shape set consisted of 8 distinct shapes and 6 distinct colors. All stimuli used in the experiment are presented in Multimedia Appendix 1 [4]. For each trial, 3 shapes and 3 colors were randomly selected without replacement to form 3 unique shape-color pairings. In the study array, these 3-colored shapes were presented within a 3×3 grid layout (9 possible positions), and each shape was randomly assigned to one of the selected locations. After a brief 900-ms delay period, either a 1-colored shape or 3-colored shapes reappeared (probe type) at different locations throughout the test array, as illustrated in Figure 1. The test arrays either maintained the same combinations of color and shape (a *same* trial) or two of the shapes swapped colors (a *different* trial). The 3 selected color and shape elements remained constant within each trial, only their pairings were changed. Participants indicated whether they believed the combination of shapes and colors presented in the test array match the combinations presented in the study array by pressing the *same* or *different* buttons located at the bottom of the screen. Test array stimuli remained on the screen for either a short (3000 ms) or long (10,000 ms) period (response duration). Each trial has either a 50% to 50% chance or 20% to 80% chance (probability of change) of being a *same* or *different* trial.

All manipulations were implemented within-persons across 8 consecutive days of ambulatory testing. Each participant completed 2 versions of the task per day, with 60 trials per version, culminating in up to 960 total trials across the 8 days. The 2 task versions were administered according to a randomized schedule. To evaluate testing experience and assess potential strategy use, participants completed a free response debrief item at the end of the study. The type of probes used at testing (all 3 items presented simultaneously in the whole-display format or a single item presented in the single-probe format) may have enabled the use of cognitive strategies such as focusing on a subset of items or using self-generated mnemonic heuristics. Instructions did not explicitly prohibit strategy use (eg, verbal encoding) to avoid inadvertently increasing its salience, but we assessed for potential strategy use during the poststudy debrief. Overall, our goal was to translate the Color Shapes tasks for unsupervised, ambulatory use while evaluating task designs suitable for extracting latent cognitive parameters through drift diffusion modeling, described in the next section.

#### Debrief Survey

Upon completing the final session, participants completed the exit survey consisting of 3 items. First, participants were asked how typical their routine, activities, and experiences were during the 8 study days on a 5-point scale, where 1=very unusual, 2=unusual, 3=neutral, 4=typical and 5=very typical, with a prefer not to respond option. Second, participants were asked whether they were impacted by any unusual circumstances or stressful events over the study duration. There were 13 response options ranging from nothing unusual to various stressful events (eg, negative social interaction, health issues, financial issues, and world events). Finally, participants were asked, “Over time, did you develop any strategies for better performance on the brain games?” (yes or no). If they responded yes, they were asked to provide more details about the strategies they used. This was an unstructured free response question. We only analyzed responses from the third item on cognitive strategy use in this study.

### Computational Modeling With the DDM

**Figure 2:**
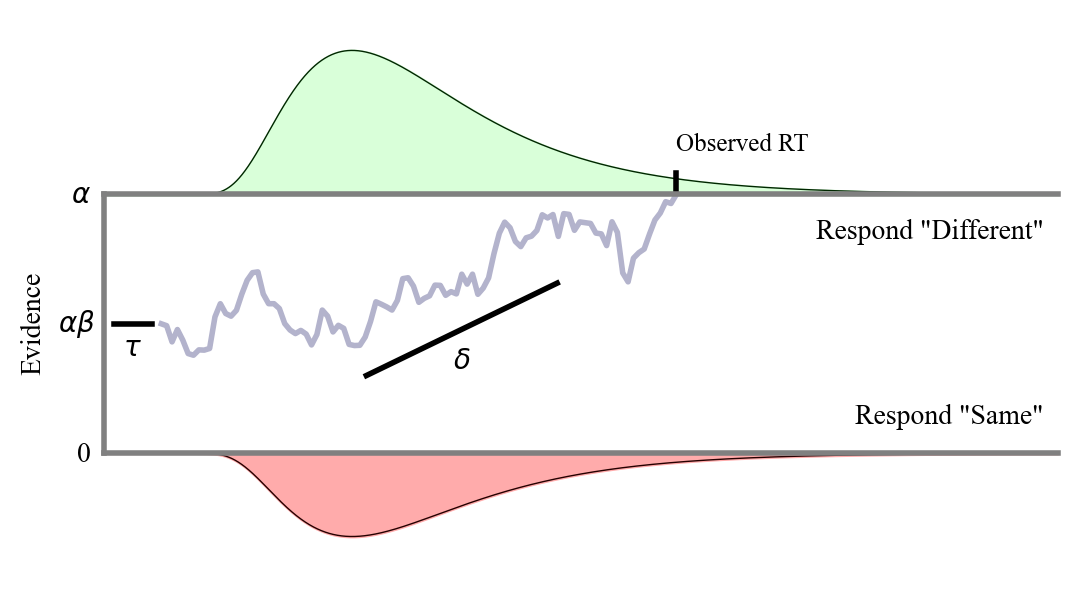
An illustration of the drift diffusion model. In a decision-making process, evidence is accumulated over time at an average drift rate of δ. The decision process terminates if the cumulative evidence value reaches 0 (lower boundary for "respond Same") or α (upper boundary for "respond Different"). At the onset of a trial, the decision process can be biased due to the amount of preliminary evidence given by αβ. The nondecision time τ reflects the duration of perceptual processes such as stimulus encoding and response execution, which occur outside the actual decision-making process. Equation 1 in Multimedia Appendix 2 describes the response time (RT) distributions that follow from these model assumptions. Figure generated via [39].

The DDM falls under the family of sequential sampling models with continuous time and continuous evidence [32]. A graphical illustration of the model is shown in Figure 2. This model assumes that each decision-making task starts a stochastic information accumulation process of incoming evidence toward one of the two response options**.** The observed decision is determined by the process terminating at one of the decision boundaries, represented in Figure 2 as the upper (*respond Different*) and lower (*respond Same*) bounds of the Color Shapes task. The distance between these bounds (the boundary separation) captures the threshold amount of evidence needed for a response to be chosen. Mathematically, the information accumulation process is modeled as a 1D or Wiener diffusion process that terminates at the absorbing response boundary. With evidence toward the upper boundary represented by positive values and lower boundary represented by negative values, decisions are based on this Wiener diffusion process in continuous time as a single total that integrates both antagonistic types of evidence.

The 4 core parameters of the DDM are presented in Table 1 (adapted from the studies by Ratcliff and Vandekerckhove [32,38]). Formally, the drift rate (*δ*) describes the speed of information accumulation by capturing the average amount of evidence that is accumulated from the stimulus over a small period, the boundary separation (*α*) describes the level of evidence required to make a decision (capturing speed-accuracy trade-off), the initial bias (*β*) describes the starting status toward one or the other boundary before the evidence accumulation process, and the nondecision time (*τ*) represents the component of RT that is not related to the decision-making process. When an individual is presented with 3 colored shapes in the study array across repeated trials, the DDM models the evidence accumulation process toward a *different* or *same* decision using the individual’s initial bias, nondecision time, boundary separation, and evidence accumulation rate. More details on the mathematical formulation of the model are provided in Multimedia Appendix 2 [17,39-46]. Given a participant’s choice RT data, we can estimate these parameters and interpret them in the context of the Color Shapes task [38].

**Table 1:** Summary of the parameters of the drift diffusion model.

Symbol	Parameter	Definition	Interpretation
*δ*	Drift rate	Average quality of the stimulus and information accumulation process	Higher *δ* indicates faster accumulation of evidence; lower *δ* indicates slower accumulation
*β*	Initial bias	Starting point bias for either response	*β*>.5 indicates bias toward *different* response; *β*<.5 toward *same*
*α*	Boundary separation	Distance quantifying evidence required to make a decision; speed-accuracy trade-off	Higher *α* indicates more caution and higher accuracy; lower *α* indicates reduced caution and lower accuracy
*τ*	Nondecision time	Motor response time, encoding time	High *τ* indicates slow encoding or motor response; low *τ* toward faster encoding

In a decision-making process, evidence is accumulated over time at an average drift rate of δ. The decision process terminates if the cumulative evidence value reaches 0 (lower boundary for *respond Same*) or α (upper boundary for *respond Different*). At the onset of a trial, the decision process can be biased due to the amount of preliminary evidence given by αβ. The nondecision time τ reflects the duration of perceptual processes, such as stimulus encoding and response execution, which occur outside the actual decision-making process. Equation 1 in Multimedia Appendix 2 describes the response-time distributions that follow from these model assumptions. Figure generated via the study by Chavez et al [47].

### Design and Procedure

After providing informed consent and enrolling in the study, participants were sent smartphones configured with the Color Shapes cognitive assessments. Next, a remote meeting with a study administrator was scheduled where they were onboarded onto the study and trained on the study protocol (eg, smartphone instructions). Over the next 8 consecutive days, participants were instructed to complete a self-initiated daily session consisting of overall 16 versions of the Color Shapes task (see description below), with each daily session taking <20 minutes. Two versions of the task were completed once per day over 8 days, and the order of the presented 16 versions were drawn randomly from 3 different schedules. Each version consisted of 60 ultrabrief trials per task, with a maximum of 960 total possible trials. The participants could only complete the testing once per day, the testing had to be completed in a single setting, which timed out after 30 minutes. Make-up opportunities were not provided for missed or incomplete sessions, and participants were presented with the next scheduled versions. Reminder alerts were provided every hour until 9 PM. Upon completing the final session, participants completed a final exit survey on the smartphone. Afterward they were debriefed by a study coordinator via videoconference and were provided with instructions to return the smartphone.

We experimentally manipulated features of the Color Shapes task in line with prior parameter selection experiment in cognitive modeling. These included four within-person variables in the Color Shapes task: (1) study time, (2) probability of change, (3) choice urgency, and (4) probe types.

We had 2 objectives in mind: to test the sensitivity for capturing changes in terms of the latent parameters of the DDM and to compare the 3-colored shapes test array and 1-colored shape test array versions of the task shown in Figure 1. Specifically, we manipulated across four task features, each with 2 levels:

1. Short (500 ms) versus long (2000 ms) study array time
2. Low (50% *different* trials and 50% *same* trials) versus high (80% *different* trials and 20% *same* trials) proportion of *different* trials, capturing different probabilities of change in the properties of the shapes from the study array to the test array
3. Minimal choice urgency (10,000 ms to respond) versus high choice urgency (3000 ms to respond)
4. Whole display (3 colored shapes simultaneously presented in the test array) versus single probe (1 colored shape simultaneously presented in the test array)

Previous cognitive modeling research has demonstrated that each of these manipulations map onto distinct parameters of the DDM [48,49]. Drift rate, the speed of information accumulation, is enhanced by longer study times as it enables more effective encoding and speed [50,51]. Adjusting the probability of change shapes the initial bias point of decision-making, shifting it toward the more frequently occurring response [34]. Manipulating response urgency influences boundary separation, mapping onto the speed accuracy trade-off where increased urgency leads to more impulsive responses and has been found to be more prevalence with age [50]. Finally, single-probe designs have been shown to effectively isolate feature-binding processes in visual working memory targeted by the Color Shapes task that has been identified as an early cognitive marker of preclinical AD [6], alongside broader utility in assessing individuals with diminished memory capacity [52]. Accordingly, we designed each experimental manipulation to target a distinct DDM parameter and evaluate its sensitivity in an ambulatory, unsupervised testing context (Table 2).

**Figure 3:**
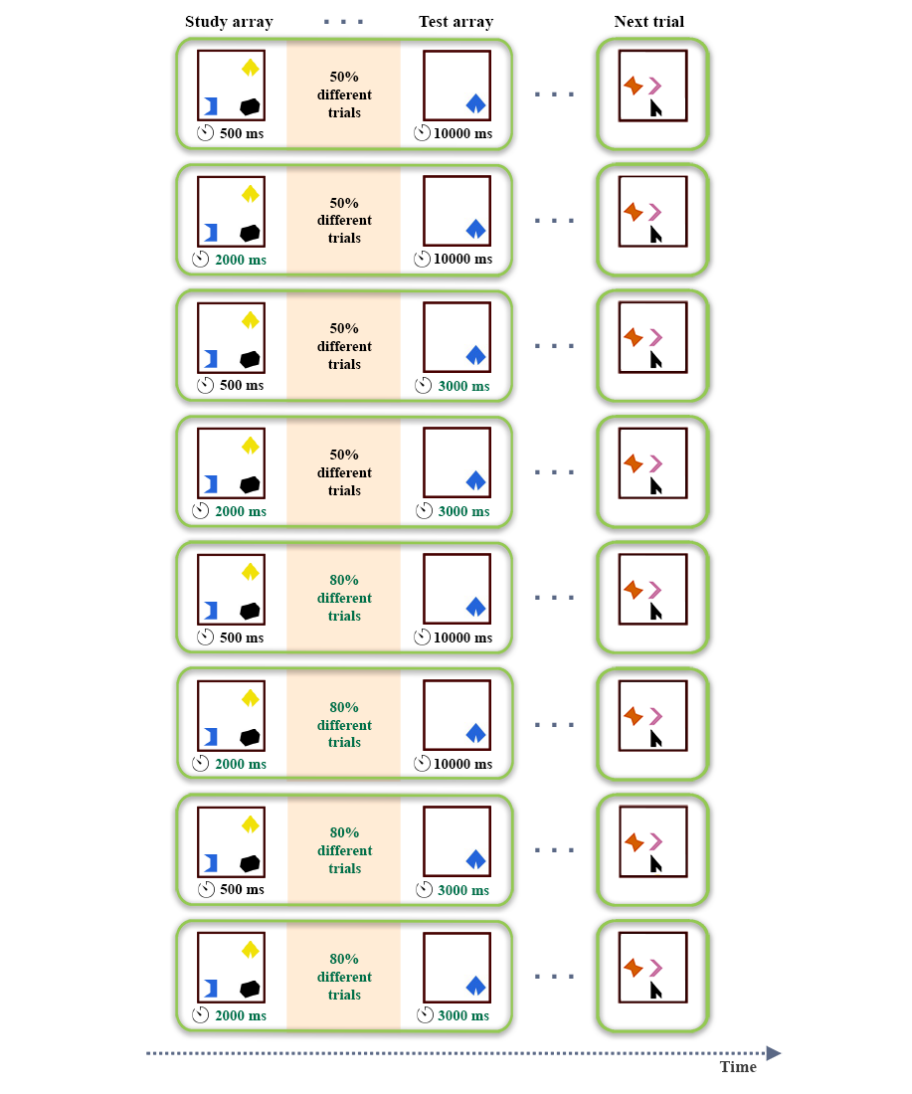
An illustration of 8 out of 16 Color Shapes task versions (all with test arrays with 1 colored shapes) with examples of different trial types shown here. Manipulations for task arrays are emphasized in green font and baseline conditions in black font. Versions have been ordered to flow from least manipulated to most manipulated versions.

Manipulations (1-3) aimed at influencing parameters of the DDM: (1) drift rate (*δ*) with the study array, (2) initial bias (*β*) with the different probabilities of change, and (3) boundary separation (*α*) with the different time urgency to respond. The nondecision time (*τ*) was estimated across participants and conditions, but we did not make any predictions about possible differences in it across conditions. Experimental manipulations were not communicated to participants. An illustration of these 3 cognitive task manipulations is shown in Figure 3 for the single probe trials.

We had the following hypotheses about the effects of the manipulations on the parameters:

1. We expected faster drift rates with longer study times, that is, more time for encoding leading to faster accumulation of evidence
2. Initial bias toward *different* responses with higher probability of *different* trials than the 50% to 50% probability
3. Lower (closer) boundary separation, that is, less caution in decision-making with increased urgency to choose a response
4. We studied how the first 3 manipulations would work with 2 visually different versions of the task, where the test array with single probe version has the additional advantage of reducing unwanted strategy use.

The experimental manipulations are summarized in Table 2.

**Table 2:** Drift diffusion model experimental manipulations and hypothesized effects.

Condition, parameter, and levels			Hypotheses
**Study time**			
	**Drift rate (*δ*)**		
		500 ms study time (short study time)	Slower drift
		2000 ms study time (long study time)	Faster drift
**Probability of change**			
	**Initial bias (*β*)**		
		50% *different* or 50% *same* (low change)	No bias
		80% *different* or 20% *same* (high change)	Bias toward *different*
**Choice urgency**			
	**Boundary separation (*α*)**		
		10,000 ms response-time window (minimal urgency)	Farther boundaries
		3000 ms response-time window (high urgency)	Closer boundaries
**Probe type**			
	Test array with 3 colored shapes (whole display)		No expected effects
	Test array with 1 colored shape (single probe)		No expected effects

### Data Processing

We set the RT-based outliers attending to the real-world constraints of the Color Shapes task. The DDM was intended to assess decision-making for fast 1-shot processes. Trials with RTs faster than 200 ms and slower than 7000 ms were excluded. Ultrafast RTs suggested technical errors, and long RTs were assessed as not following speeded task instructions or not paying attention to the task.

### Data Analysis

We fit a tailored, multilevel DDM to RT and accuracy to derive latent cognitive parameters of interest. The multilevel DDM was cast in a Bayesian framework [41]. We estimated drift rates, initial bias, boundary separation, and the non–decision time parameter for each condition and individual, allowing these parameters to vary between all 16 conditions with no constraints. Finally, the RT data were scaled from milliseconds to seconds for the analysis. A detailed description of our multilevel DDM is provided in Multimedia Appendix 2. All analysis scripts and data are provided on the Open Science Framework [39].

## Results

### Participant Characteristics

We recruited 69 participants for the study but excluded 1 participant due to missing 99.1% of data (9/960 trials completed). Therefore, our final sample size was 68, and demographics for these participants are displayed in Table 3. The average age of participants was 49 years; 69% (47/68) were women, 31% (21/68) were men, and none identified differently. Participants’ ages spanned across younger to older adulthood (mean 49, SD 14; range 24-80 years), as visualized in Multimedia Appendix 3. Regarding race, 81% (55/68) identified as White, 4% (3/68) as Black or African American, 6% (4/68) as Asian, 1.5% (1/68) as American Native or Alaska Native, 1.5% (1/68) as Native Hawaiian or Pacific Islander, 3% (2/68) as multiethnic, and 3% (2/68) as other. As for ethnicity, 96% (65/68) identified as non-Hispanic and 4% (3/68) identified as Hispanic. In terms of highest education received, 5% (3/68) reported high school, 14% (9/68) reported vocational or some college, 12% (8/68) received an associate’s degree, and 68% (46/68) reported at least a bachelor’s degree or higher.

**Table 3:** Demographic characteristics of the sample (N=68).

		Values
**Gender, n (%)**		
	Women	47 (69)
	Men	21 (31)
**Race, n (%)**		
	Asian	4 (6)
	American Native or Alaska Native	1 (1)
	Black or African American	3 (4)
	Multiethnic	2 (3)
	Native Hawaiian or Pacific Islander	1 (1)
	Other	2 (3)
	White	55 (81)
**Ethnicity, n (%)**		
	Non-Hispanic	65 (96)
	Hispanic	3 (4)
**Education, n (%)**		
	High school	3 (5)
	Vocational or some college	9 (14)
	Associate’s degree	8 (12)
	Bachelor’s degree	19 (29)
	Post–bachelor’s degree	2 (3)
	Master’s degree	19 (29)
	Doctoral degree	6 (9)
	N/A^a^	2 (3)
Age (y), mean (SD); range		49 (14); 24-80

^a^N/A: not applicable.

### Descriptive Statistics

In total, 41 participants completed all possible 960 trials (16 versions by 60 trials); 22 participants completed at least 75% (720/960) of the trials, and 4 participants completed 62.5% (600/960), and only 1 participant completed only 25% (240/960) of the trials. A summary of participant-level trial engagement is shown in Table 4. The overall rate of unrecorded or missing trials was 8.45% (5,518/65,280). See Multimedia Appendix 4 for a detailed breakdown of trial exclusion percentages. In short, from the 65,280 total possible trials, 59,762 (91.55%) trials were recorded, and a total of 58,978 (90.35%) trials were retained for analysis. In total, 784 trials (1.20%) were excluded based on predefined criteria. Among these, 720 (1.10%) were removed because they exceeded the allowed response window in the choice urgency condition, and 54 trials (0.09%) were removed due to unrealistic values (eg, unrealistically fast RTs <200 ms or slow RTs >7000 ms).

Table 5 shows that accuracy rates by test array condition (single probe vs whole display). The mean overall accuracy rate of correct trials among all trials was 85.83% (50,623/58,978). Accuracy rate was higher in the whole display (26,945/29,427, 91.57%) than in the single probe (23,678/29,551, 80.13%) condition.

**Table 4:** Summary on participant engagement during the study (N=68).

Completion status, %	Trials completed, n	Versions completed, n	Participants, n (%)
100.0	960	16	41 (60)
93.8	900	15	2 (3)
87.5	840	14	13 (19)
75.0	720	12	7 (10)
62.5	600	10	4 (6)
25.0	240	4	1 (2)

**Table 5:** Accuracy rates and response times across all trials and between test array probe types.

Probe type	Correct trials, n	Total trials, n	Accuracy, %	Response time (ms)
Overall	50,623	58,978	85.83	1290.95
Whole display	26,945	29,427	91.57	1314.97
Single probe	23,678	29,551	80.13	1270.35

### Model Fit

**Figure 4:**
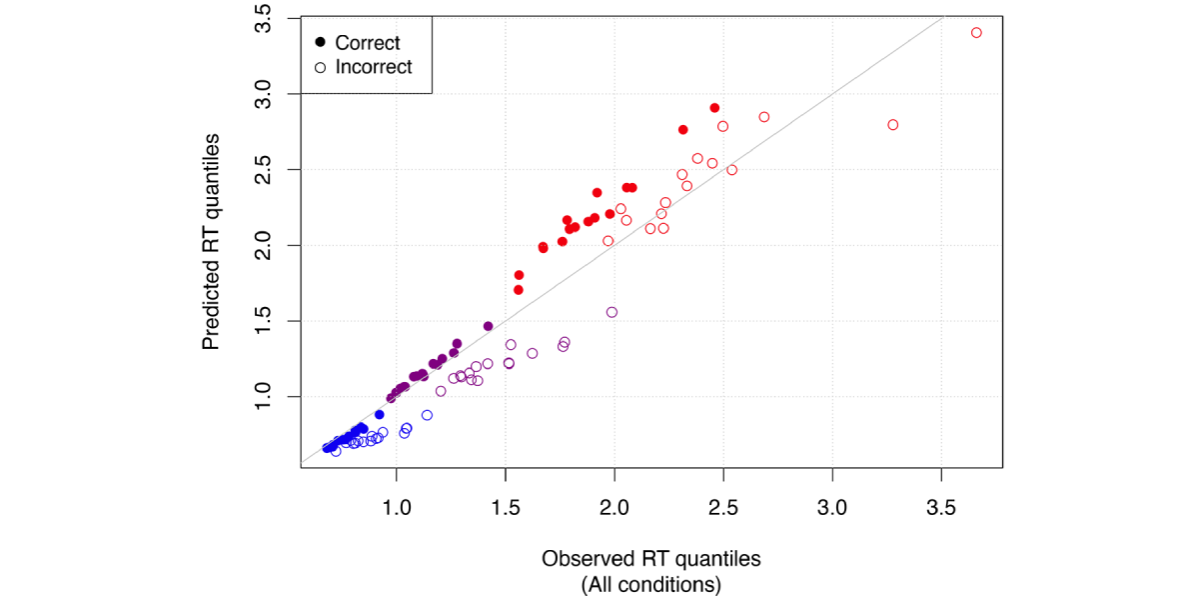
Posterior predictive check for all 16 conditions. RT: response time.

**Figure 5:**
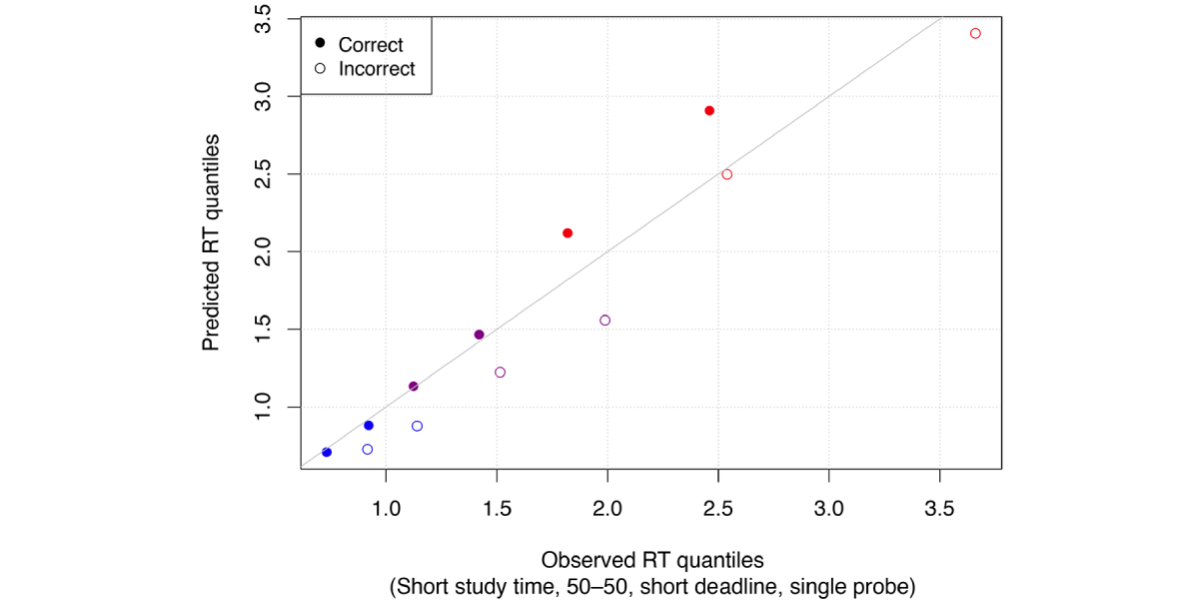
Posterior predictive check for conditions (short study time, 50%-50% distribution of trials, high urgency, single probe). RT: response time.

To evaluate model fit, we simulated posterior predictive data (n=6000) based on the DDM. In Figure 4, we plotted observed (data-based) and model-predicted RT quantiles for all 16 conditions. Empty circles represent quantiles of incorrect response RT distributions, and filled circles are correct responses. We can see that the observed and model predicted values line up well for RTs below 2 seconds, with more variance occurring after 2 seconds. However, even for these larger RTs, the fit is reasonable. In Figure 5 we only show these for a single probe version, with 500-ms study time, no choice urgency, and half *different* or half *same* probes (our recommended constellation of features for future use in combination with drift diffusion modeling, see the discussion section). This plot also shows great fit with some flaring for slow RTs, which are more variable, and some underestimation of error RTs, which are only a small proportion of the data.

### Group-Level Summaries of DDM Parameters

Summaries of DDM parameter estimates are shown for the condition that was aimed for their manipulations in Table 6. Results are separated for the different probe type test array conditions: whole display and single probe. Mean and 95% credible interval estimates are based on the posterior distribution of the corresponding parameters. The credible interval is a Bayesian statistic that can quantify the uncertainty that the true parameter value lies within the interval. A 95% credible interval indicates that the interval has a 95% probability of containing the true value. If 0 is included in the 95% credible interval, we conclude that there is no credible evidence that the parameter is different from 0.

For clarity, findings are presented similarly in structure across the three DDM manipulations: (1) drift rate is displayed with the 2 conditions related to study time, (2) initial bias is shown with the probability of change conditions, and (3) boundary separation is shown with choice urgency conditions.

For drift rate, higher absolute *δ* values represent faster accumulation of evidence. By definition, the initial bias ranges with *β* values between 0 and 1, with a *β*=.5 indicating no bias. For the ease of regression model (initial bias estimates were regressed on task conditions), we worked with logit transformed initial bias estimates (see details in Multimedia Appendix 2), which meant that positive estimated *β* values expressed bias toward *different* response, while negative *β* values for *same* response. When the corresponding 95% credible interval contained 0, we concluded that there was no credible bias in either way (which was expected for the condition with equal proportion of *same* and *different* trials). Finally, as the boundary separation parameter by definition could only take positive values, we applied a log-transformation to this parameter to ease regression modeling. Higher *α* values simply relate to farther response boundaries in the log scale as well, just like in the original scale.

While we can see clear differences in Table 6 between the conditions in both probe types, we need to test whether these differences are credible. Therefore, for the manipulated features listed in (1-3), we estimated contrast parameters by taking the difference between each condition (manipulation—baseline) to examine differences on expected cognitive processes. Specifically, we estimated 3 contrast parameters: study-time-contrast (difference in long study time—short study time), probability-of-change-contrast (high change—low change), and choice-urgency-contrast (high urgency—minimal urgency). The Bayesian framework allowed us to derive these contrasts as regular model parameters with posterior distributions, which allows for principled testing of our hypothesis. Table 7 summarizes the estimates for these below. All parameters were estimated simultaneously within a single-step model estimation process. The strength of this approach is the correct carryover of uncertainty and avoiding bias in sequential estimations [53]. We discuss results for each DDM parameter manipulation next.

**Table 6:** Posterior summaries of the drift diffusion model parameters per conditions^a^.

Parameter, condition, and probe type			Posterior mean (SD)		95% credible interval
**Drift rate (*δ*)**					
	**Long study time^a^**				
		Whole display	1.37 (0.12)	1.13 to 1.58	
		Single probe	0.90 (0.13)	0.66 to 1.12	
	**Short study time**				
		Whole display	1.65 (0.18)	1.37 to 2.00	
		Single probe	0.88 (0.09)	0.70 to 1.06	
**Initial bias (*β*)**					
	**High change^a^**				
		Whole display	0.13 (0.03)	0.07 to 0.20	
		Single probe	0.10 (0.04)	0.02 to 0.18	
	**Low change**				
		Whole display	0.07 (0.04)	−0.02 to 0.14	
		Single probe	−0.05 (0.09)	−0.19 to 0.10	
**Boundary separation (*α*)**					
	**High urgency^a^**				
		Whole display	0.91 (0.09)	0.78 to 1.05	
		Single probe	0.66 (0.07)	0.56 to 0.77	
	**Minimal urgency**				
		Whole display	0.92 (0.13)	0.73 to 1.12	
		Single probe	0.71 (0.08)	0.59 to 0.87	

^a^Manipulation condition.

**Table 7:** Posterior differences of manipulated condition on drift diffusion model parameters.

Parameters, condition, and probe type				Posterior mean (SD)		95% credible interval
**Drift rate (*δ*)**						
	**Long study–short study**					
		Whole display	−0.28 (0.05)		−0.39 to −0.18	
		Single probe	0.01 (0.05)		−0.09 to 0.11	
**Initial bias (*β*)**						
	**High change–low change**					
		Whole display	0.06 (0.02)		0.02 to 0.10	
		Single probe	0.15 (0.02)		0.11 to 0.20	
**Boundary separation (*α*)**						
	**High urgency–minimal urgency**					
		Whole display	−0.01 (0.02)		−0.04 to 0.03	
		Single probe	−0.04 (0.02)		−0.08 to −0.01	

### Study Time on Drift Rates

First, we examined the differences in drift rate estimates (*δ*) between the study time conditions, broken down by probe type conditions (upper part of Table 7). For the posterior means of the study-time-contrast parameter (difference in long study time–short study time), positive difference values indicate faster drift rates in the manipulated long study time (2000 ms) than in the control short study time (500 ms), while negative difference values indicate slower drift rates in the manipulated condition compared with the baseline condition. As can be seen from Table 7, the drift rate was credibly higher in the control condition in the whole display probe version of the task. While the manipulation had no credible effect in the single probe version of the task.

**Figure 6:**
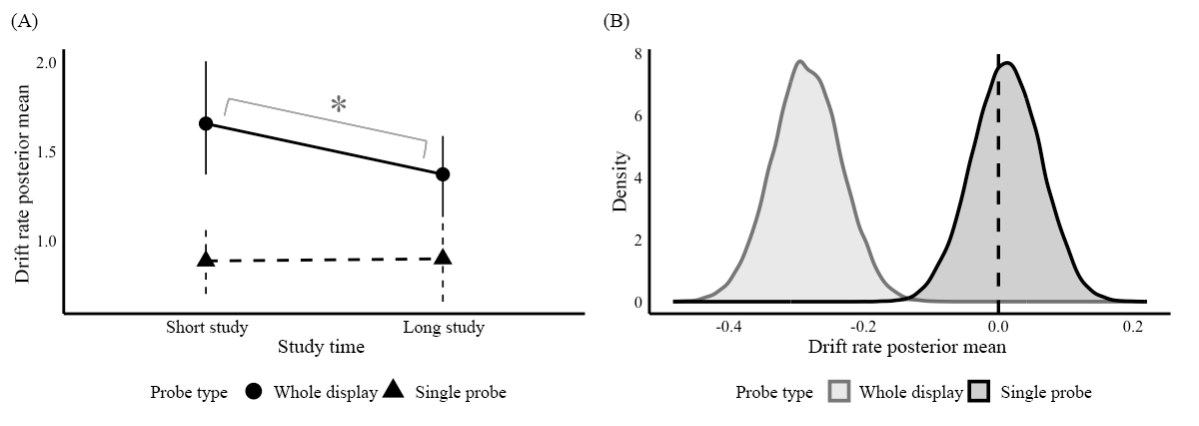
Posterior means and posterior differences of drift rate parameters for study time conditions. (A) Drift rate posterior group means; (B) Distribution of drift rate posterior differences.

Our drift rate manipulation did not show the hypothesized effect. We expected faster drift rates when participants were allowed more time to study the visual features; however, we observed no credible change for single probe and slower draft rates as well. Figure 6A illustrates the posterior mean summaries of the estimated average drift rate between study time conditions for the different probe types (see the values in Table 6). Drift rates were credibly faster on whole display trials with shorter time to inspect the study array (baseline condition: mean 1.65, SD 0.18; 95% credible interval 1.37-2.00) than when given more time to study (manipulated condition: mean 1.37, SD 0.12; 95% credible interval 1.13-1.58). This is also illustrated in Figure 6B, which shows that the light gray posterior distribution of the drift rate differences (study-time-contrast parameter) between study time and probe type conditions (mean –0.28, SD 0.05; 95% credible interval –0.39 to –0.18) does not overlap with 0. This finding was unexpected, as we expected that longer time to study the test array would result in faster drift rates. Drift rates on single probe trials did not differ credibly between shorter study times (mean 0.88, SD 0.09; 95% credible interval 0.70-1.06) and longer study times (mean 0.90, SD 0.13; 95% credible interval 0.66-1.12). As shown in Figure 6B, the dark gray posterior distribution of the study-time-contrast parameter overlaps with 0 (mean 0.01, SD 0.05; 95% credible interval –0.09 to 0.11); that is, our drift rate manipulation simply did not influence single probe trials.

### Probability of Change Manipulation on Initial Bias

Second, we examined the estimated probability-of-change-contrast parameter between probe types. This manipulation was intended to influence the initial bias parameter (*β*) that captures the degree of a priori bias toward *different* or *same* response options. As mentioned earlier, this parameter was logit-scaled, with values at 0 representing no systematic bias toward both binary responses, positive values indicating greater bias toward the *different* response, and negative values indicating bias toward the lower *same* response. Therefore, in Table 7, for the calculated contrast parameter taking the difference in initial bias estimates between the probability of change conditions (high change—low change), the positive values indicated greater a priori biases toward the *different* response boundary in the manipulated high change condition.

**Figure 7:**
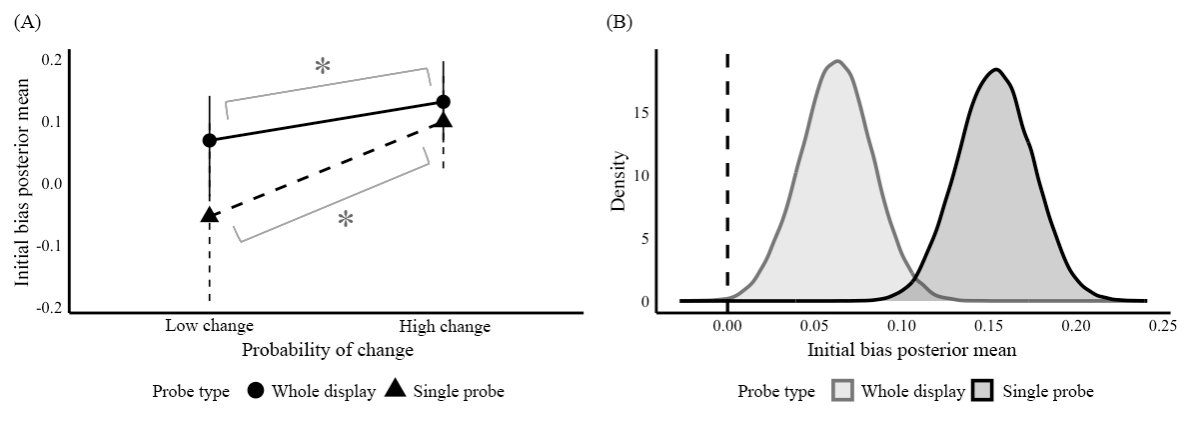
Posterior means and posterior differences of initial bias parameter for probability of change conditions. (A) Initial bias posterior group means; (B) Distribution of initial bias posterior differences.

We observed positive changes in initial bias with higher probability of change manipulation in both probe types—all contrast values were credibly positive. Figure 7A shows the posterior mean summaries of the initial bias parameter between probability of change conditions and probe types. When tasks were manipulated to have higher probabilities of *different* trials (48/60, 80% of trials) than *same* trials (12/68, 20% of trials), estimates of the initial bias were sensitive to the manipulation and took positive values overall, reflecting a tendency toward selecting the *different* boundary, both on whole display trials (mean 0.13, SD 0.03; 95% credible interval 0.07-0.20), and in single probe trials (mean 0.10, SD 0.04; 95% credible interval 0.02-0.18). As expected, trials with even presentation of *different* and *same* stimuli indicated negligible response bias in both whole display (mean 0.07, SD 0.04; 95% credible interval –0.02 to 0.14), and in single probe trials (mean –0.05, SD 0.09; 95% credible interval –0.19 to 0.10), as shown in Table 5.

The initial bias manipulation effect is further illustrated in Figure 7B, where the distribution of posterior differences in *β* per probability of change conditions are shown. The range of the contrast parameter’s distributions for both probe types do not include 0, suggesting credible effects of this manipulation on the initial bias parameter across probe types. This effect is more pronounced in the single probe condition (dark gray distribution: mean 0.15, SD 0.02; 95% credible interval 0.11-0.20) than the whole display condition (light gray distribution: mean 0.06, SD 0.02; 95% credible interval 0.02-0.10).

### Choice Urgency Manipulation on Boundary Separation

Third, we examined differences in the boundary separation parameter by choice urgency and probe type conditions. This manipulation was intended to influence the response boundary separation: the amount of evidence required to make a decision. Larger boundary separation corresponds to higher threshold values (*α*). We expected the boundary separations to be lower in the manipulated condition with higher choice urgency to respond (3000 ms) than in the baseline condition with minimal urgency and more time to respond (10,000 ms). The boundary separation parameter was log-transformed to ease the regression modeling, but this scale remained easy to interpret as higher boundary separation values still indicated more information needed before making a decision (with values closer to 0 indicating less information acquired before making a decision). In Table 7, negative choice-urgency-contrast values (high urgency-minimal urgency) indicated lower boundary separation in the manipulated condition (high urgency). Positive differences represented higher response boundary separation in the manipulated high urgency condition than the control minimal urgency condition.

**Figure 8:**
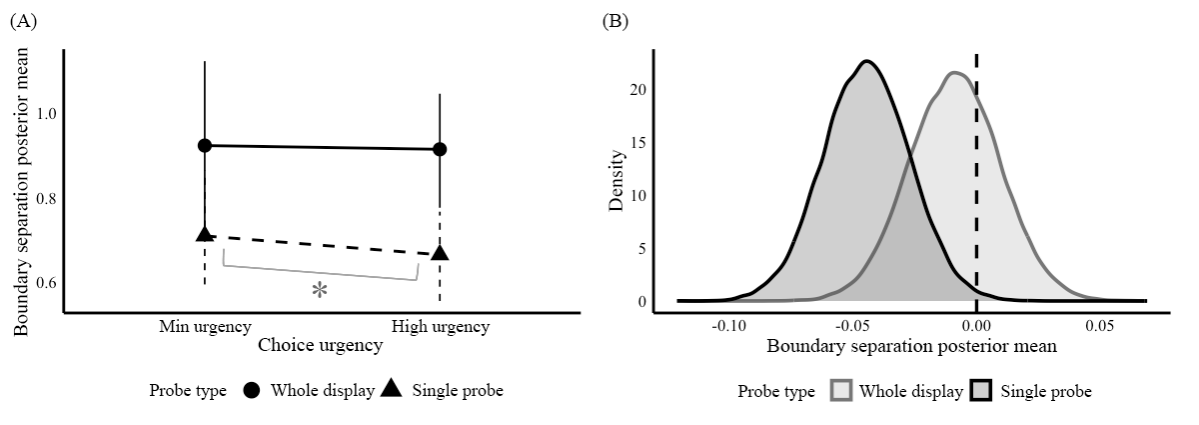
Posterior means and posterior differences of the boundary separation parameter for choice urgency conditions. (A) Boundary separation posterior group mean; (B) Distribution of boundary separation posterior differences.

Our boundary separation manipulation worked as expected for the single probe version of the task. The posterior mean summaries of the boundary separation parameter between choice urgency conditions and probe types are shown in Figure 8A, based on values from Table 6. In the single probe trials, this manipulation was successful as boundary separation parameters were sensitive to the different choice urgency conditions (mean –0.04, SD 0.02; 95% credible interval –0.08 to –0.01), with participants exhibiting lower boundary separation with heightened urgency to respond to trials. This contrast parameter for single probe trials is shown in Figure 8B, with almost all the dark gray posterior distribution being <0. Comparatively, looking at the light gray distribution corresponding to the whole display trials, we saw that 0 is almost in the middle of that distribution (mean –0.01, SD 0.02; 95% credible interval –0.04 to 0.03), signifying no credible effect for this manipulation in the whole display condition.

### Bayesian Correlations

We explored associations between age and the 3 person-specific parameters of the DDM (drift rate, boundary separation, and initial bias) by running Bayesian correlation analysis in JASP (version 0.17.3) [54]. This analysis yields a regular Pearson correlation coefficient for each tested link and a corresponding Bayes factor that in our case summarizes the amount of evidence for the coefficient to be different from 0 [15]. For instance, Bayes factors of (*BF_10_*) 3 to 10 indicate moderate evidence for the correlation, 10 to 30 indicate strong evidence, 30 to 100 indicate very strong evidence and beyond. On the basis of a previous study [24], we predicted that older participants would have slower drift rates and higher boundary separation in decision-making, while we did not expect age to be credibly linked to initial bias. We tested associations across all trials then stratified by probe types. Correlation coefficients and their corresponding Bayes factors are shown in Table 8.

**Table 8:** Bayesian correlations of drift diffusion model parameters and age.

Parameters and probe type		Pearson *r*	*BF _10_* ^a^	95% credible interval
**Drift rate (*δ*)**				
	Overall	−0.45^b^	191.11	−0.61 to −0.23
	Whole display	−0.39^c^	32.33	−0.57 to −0.17
	Single probe	−0.42	76.04	−0.59 to −0.20
**Initial bias (*β*)**				
	Overall	0.21	0.62	−0.03 to 0.42
	Whole display	0.31	4.26	0.08 to 0.51
	Single probe	−0.04	0.16	−0.27 to 0.19
**Boundary separation (*α*)**				
	Overall	0.32	4.88	0.09 to 0.51
	Whole display	0.22	0.74	−0.02 to 0.43
	Single probe	0.42	80.76	0.20 to 0.59

^a^Represents ratio of likelihood of evidence in favor of alternative model (1) than null model (0).

^b^*BF*_10_>30.

^c^*BF*_10_>10.

As expected, the Pearson *r* coefficients capturing association between age and overall drift rate indicated a credibly negative association with age (*r*=–0.45; 95% credible interval –0.61 to –0.23; *BF*_10_=191.11), suggesting that older participants have slower drift rates. Results indicated extreme evidence for this association and that the data were at least 191 times more likely under the alternative hypothesis of correlation than the null hypothesis of no correlation. We found similar links when we broke down this association by probe type. The results indicated very strong evidence for moderate negative association between drift rate and age in single probe trials (*r*=–0.42; 95% credible interval –0.59 to –0.20; *BF*_10_=76.04) and whole display trials (*r*=–0.39; 95% credible interval –0.57 to –0.17; *BF*_10_32.33). As expected, we did not find evidence for association between initial bias and age across both probe type trials (*r*=0.21; 95% credible interval −0.03 to 0.42; *BF*_10_0.62). People’s age was not expected to be linked with whether they are more likely to choose *different* or *same* on a trial. However, when we separated the probe types, we found some weak evidence of association in the whole display condition (*r*=0.31; 95% credible interval 0.08-0.51; *BF*_10_=4.26). Importantly, the single probe condition showed evidence for the lack of correlation (*r*=−0.04; 95% credible interval −0.27 to 0.19; *BF*_10_=0.16)—this can be seen by taking the reciprocal of the corresponding Bayes factor (*BF*_10_=1/0.16=6.25), which represented 6 times more evidence for the lack of correlation.

The Pearson *r* coefficients showed a positive association between age and boundary separation (*r*=0.32; 95% credible interval 0.09-0.51; *BF*_10_=4.88). This suggested that older participants had higher boundary separation. Across all probe type trials, and the Bayes factor indicated moderate evidence and that the data were at least 4 times more likely under the alternative hypothesis of correlation than the null hypothesis of no correlation. Importantly, this boundary separation association was driven by the single probe trials. The correlation between boundary separation and age in single probe trials was *r*=0.42 (95% credible interval 0.20-0.59; *BF*_10_=80.76), with very strong evidence. For the link between age and boundary separation in whole display, we did not find a credible association and the Bayes factor provided no evidence (*BF*_10_≈1) for the alternative hypothesis (*r*=0.22; 95% credible interval –0.02 to 0.43; *BF*_10_=0.74).

### Strategy Use

Subsequently, we conducted a post hoc qualitative analysis of cognitive strategy use in light of the unexpected effect of study time on drift rates. As part of the debrief and exit survey, participants were presented with a brief series of unstructured questions. Summary of strategy use is presented in Table 9, and all the raw responses are reported in Multimedia Appendix 5. From the 68 participants, 81% (n=55) reported using some strategy, whereas 13% (n=9) reported no strategy used. Individuals who responded “Yes” were presented with an open-ended response to elaborate on specific strategies. One common theme emerged upon reviewing the responses. Many strategies involved using some tactic involving ≥2 shapes. Responses were categorized based on whether individuals reported any mention of 2 shapes or not. Approximately 60% (n=33) reported some strategy using 2 shapes, whereas the other 40% (n=22) individuals reported a different strategy that did not involve 2 shapes. For instance, participants reported using mnemonic strategies, such as organizing objects by size, creating heuristics with objects, or integrating the array into a single object.

**Table 9:** Summary of responses related to cognitive strategy use (N=68) and post hoc analysis of reported cognitive strategy use.

Cognitive strategy reported			Responses, n (%)
**Used some strategy**			55 (81)
	Description includes 2 shapes	33 (60)	
	Any strategy that does not include 2 shapes	22 (40)	
Did not report any strategy			9 (13)
No response			4 (6)

## Discussion

### Overview

We explored the feasibility of fitting a computational model (ie, the DDM) to ambulatory Color Shapes data by experimentally manipulating features of the Color Shapes task. Our proposed approach can integrate theory-based working memory measures with computational cognitive methodology to capture computational model–based metrics of cognitive ability in daily life settings. This approach was powered by the Bayesian statistical engine, which allowed for simultaneous estimation of all latent features, as well as person- and condition-specific variations in it.

Overall, the results of this study revealed several patterns that emerged consistently across conditions. The hierarchical Bayesian model provided a good fit to the data, confirmed via posterior predictive checks. Of the 3 experimental manipulations, the initial bias and response boundary conditions effectively shifted the corresponding cognitive parameters in the single probe condition. However, the drift rate manipulation effect did not work for the single probe condition and had the opposite effect in the whole display condition. In terms of individual differences in DDM parameters, age was meaningfully associated with drift rate and boundary separation, as was expected based on previous studies on aging [55,56].

### Principal Findings

We presented a novel ambulatory adaptation of the Color Shapes task. The Color Shapes task has previously been established in the literature as a sensitive and specific measure of symptomatic and asymptomatic preclinical AD [6,7,25]. RT and accuracy data from this task have not been analyzed previously in a drift diffusion modeling framework. Our approach focused on disentangling subtle cognitive processes underlying manifest Color Shapes task performance with the DDM.

Specifically, we aimed to optimize the Color Shapes task for drift diffusion modeling in ambulatory assessment settings. Models, such as the DDM can incorporate speed and accuracy characteristics in RT data that may offer novel insights into cognitive changes that account for the speed-accuracy trade-off [14]. For this, we tested whether we are able to experimentally manipulate 3 parameters of the DDM by changing features of the Color Shapes task. We compared whole display and single probe versions across these manipulations. We also tested whether individual-level DDM parameters were meaningfully associated with participant’s age, given previous studies showed that older participants tended to have declines in speed of cognitive processing [6] and monitor responses to prioritize accuracy over swift responses [57]. Finally, we looked at whether participants would be likely to use strategies for performing better on the Color Shapes task.

Our results showed that 2 Color Shapes property manipulations affected cognitive processes as we expected, as captured by the DDM parameters. First, when we changed the proportion of *different* versus *same* trials toward *different*, the initial bias shifted, indicating subtle preference toward the *different* response in both single probe and whole display versions of the task. Second, when we manipulated the boundary separation feature by triggering choice urgency, we observed the expected effect only in the single probe version, with practically no effect in the whole display version. In the single probe condition, imposing a higher choice urgency (ie, 3000 ms response window before autoadvancement) resulted in a lower response caution, as indicated by a closer boundary separation, relative to the minimal urgency condition with a longer response window (10,000 ms). However, this manipulation did not show this similar effect in the whole display condition.

Our results showed that the presentation duration manipulation did not yield the hypothesized effect on drift rate. Specifically, increasing the amount of time to study the array did not increase the drift rate in the single probe version but produced slower drift rates in whole display trials. While our decision to include this manipulation was based on previous studies using similar methods [48], and making allowances for the difference between laboratory studies and our ambulatory setting, it is likely that our short 500 ms duration already put most subjects at ceiling level for presentation duration (see the study by Ratcliff [58] for the equivalent finding in a laboratory setting). Successfully manipulating the drift rate for the Color Shapes task in question might require a different approach, possibly making use of visual masking. Indeed, Smith [59] argues that the critical factor distinguishing whether presentation duration manipulation works is whether or not backward masks are used to limit the information extracted from the display. Note, however, that this failure to find the hypothesized effect does not otherwise speak to the validity of the study’s premise of testing the DDM, whose parameter estimates appropriately reflect the pattern in the data.

Altogether, we conclude that the single probe version of the Color Shapes task is better optimized for capturing DDM-based cognitive process dissociations in ambulatory settings than the whole display version. The single probe version might also more effectively activates a single decision-making process, in line with DDM assumptions [34]. In contrast, the whole display version may engage multi-strategy decision processes, which could obscure the attribution of cognitive shifts and diminish the validity of model parameter estimates [60]. In addition, the correlations analysis between individual-level DDM parameters and age was more consistent with the single probe version: (1) there was evidence for links between age and drift rate, which is in line with previous findings and (2) evidence for the lack of correlation between age and initial bias, as expected [61,62]. The whole display version did not show evidence for correlation between age and boundary separation and had some evidence for unexpected weak correlation between age and initial bias. Post hoc qualitative data analysis showed that most of the unwanted strategy use was related to encoding only 2 shapes out of the 3 in the study array. This strategy would not be feasible for participants in the single probe version of the task.

Our results indicated that this ambulatory task version (single probe version of the Color Shapes task with short study time [500 ms], low probability of change [50%-50% *different* or *same*], and minimal choice urgency) responded well to the intended manipulations and was associated with convergent and divergent constructs as theorized. Giving 500 ms to study has been commonly used in clinical settings and might also prevent verbal encoding of the stimuli. We found that drift rates were higher in the 500-ms condition, which might support a more 1-shot decision-making style assumed by the DDM. The other two features of the task, that is, even presentation of stimuli and no strict limit on time to respond are also typical in the literature. In terms of raw data, this condition had an average reaction time of 1252.87 ms (95% central quantile range 800.43-1799.48 ms) with a 76.82% average accuracy rate (95% central quantile range 57.63%-96.63%), which are also in range of typical data modeling with DDM. These results align with broader research demonstrating that DDM parameters are sensitive to subtle cognitive variations associated with aging, AD, and a range of neuropsychiatric conditions, including obsessive compulsive disorder, attention-deficit/hyperactivity disorder, and Parkinson disease [63-66].

This optimization of an ambulatory version of the Color Shapes task for accessibility and computational cognitive modeling advances the development of digital tools that extract novel digital markers to empower individuals to initiate health services earlier. By implementing the DDM to disentangle ambulatory cognitive performance data into more nuanced processes underlying performance, we can open opportunities to integrate cognitive aging theoretical frameworks with early detection advances ushered in by technological advances in computational tools [67] and multi-model data [68]. Our study integrated the Color Shapes task linked with preclinical AD-related cognitive changes with computational cognitive methodology and focused on their respective methodological strengths for capturing cognitive processes in daily life settings. This approach is powered by the Bayesian statistical engine that allows a person-centered approach for quantifying individual-specific risk probabilities, for example, in terms of DDM parameter estimates.

Taken together with drift diffusion modeling, the results found that this approach was effective in measuring subtle differences in latent cognitive processes from observed performance. This approach has the potential to extract sensitive digital-based cognitive indicators that can reveal subtle cognitive decline at earlier stages of AD and related dementias. The longitudinal implementation of this approach holds promise for being able to monitor these digital cognitive features over short and long periods. On a broader scale, this approach can be effectively tailored for designing person-centered treatment plans for monitoring cognitive health status over adulthood.

### Limitations and Future Directions

Finally, this study had some limitations. First, there may be underlying bias related to participants and study selection. For instance, the population of individuals that register for research participation may be qualitatively compared with individuals that do not sign-up. Second, cognitive health status was assessed via self-reported information and may be bolstered with biological or clinical validation. Third, there may be relevant reasons for missing data on cognitive performance that can be important to address in future computerized assessment designs. Fourth, we applied fixed RT cutoffs informed by domain expertise, and recommend that future work explore alternative, more flexible approaches to managing RT outliers. Fifth, participants were not formally screened for color vision deficiencies, which may have affected performance despite the task being designed using color palettes and shape elements informed by visual accessibility guidelines [69].

As this study was conducted entirely remotely, it may require additional consideration to control for unintended cognitive strategy use. We opted to ask participants at the end regarding any cognitive strategy use, rather than providing explicit instructions that may potentially bias performance data. As the Color Shapes task is intended to assess cognitive processes underlying visual working memory [5,7], using unwanted cognitive strategies using different cognitive domains (eg, verbal encoding), the Color Shapes task could fail to measure changes in visual short-term memory system and bias results. Designs to assess subtle changes associated with cognitive impairment may benefit from conducting a priori technical optimization to identify best strategies to control for unwanted cognitive strategy use. By optimizing the task for unsupervised remote testing, this aids in designing valid and reliable ambulatory cognitive instruments.

AD has a long preclinical phase marked by neuropathological changes [70]. While early stages of preclinical AD can be detected using biomarkers of amyloidosis and neurodegeneration (eg, cerebrospinal fluid, brain imaging, and blood [71]), mobile cognitive testing offers an easily accessible early monitoring tool. Due to the nature of subtle progressive cognitive decline in AD, individuals may conflate pathological cognitive decline with normative age-related changes and consequently delay seeking timely medical care [72] or wait until symptoms have worsened [73]. One overarching goal of this study was to develop an optimized version of the Color Shapes task that is effective for unsupervised, high-frequency cognitive testing. Our results showed evidence that mobilizing smartphone technology to administer ambulatory cognitive assessments in this manner is a viable approach for sampling a representative measure of cognitive status variability in daily contexts. A recent survey reported that 70% of Americans want to know if they were at risk for preclinical AD for seeking earlier treatment, however, only 60% of Americans were reluctant to discuss mild cognitive impairment symptoms and early signs with their health care providers [73]. Commonly cited reasons included concerns with receiving incorrect diagnosis, treatment, or waiting until symptoms did not resolve. Our results were promising as single probe trials may be less cognitively loading and may support long-term adherence. This study demonstrates an effective and feasible design to address related to effective screening and earlier intervention. Finally, although this study focused on deriving cognitive process parameters using the Color Shapes task, future research should explore how these parameters relate to performance on standard neuropsychological assessments. Mapping these associations would help contextualize DDM parameters and facilitate their application in cognitive and clinical settings.

### Conclusions

Collecting data with the Color Shapes task in ambulatory settings and analyzing it using a drift diffusion modeling approach yielded digital markers of key cognitive processes. We found that the single-probe (test array with 1 colored shape) version of the Color Shapes task with 500 ms (short) study time, even presentation (50% to 50%) of *different* and *same* trials, and minimal response urgency exhibited expected performance and associations. These novel digital cognitive features could facilitate more sensitive and earlier screening of individuals for secondary AD and related dementia, enable earlier intervention, and support efforts to target modifiable risk factors [1,3].

## Appendix

Multimedia Appendix 1 The Color Shapes task shapes and color palette.

Multimedia Appendix 2 Drift diffusion model.

Multimedia Appendix 3 Age distribution of participants (N=68).

Multimedia Appendix 4 Summary of included and excluded trials.

Multimedia Appendix 5 Open-ended responses on cognitive strategy use collected during the debrief survey.

## Abbreviations

AD: Alzheimer disease
DDM: drift diffusion model
RT: response time
