# Supplementary material for: Optimizing the Color Shapes Task for Ambulatory Assessment and Drift Diffusion Modeling: A Factorial Experiment
Source: JMIR Form Res. 2025 Oct 1;9:e66300. doi: 10.2196/66300 (PMC12530164; doi:10.2196/66300)
Supplement: Multimedia Appendix 1 [file formative_v9i1e66300_app1.docx]

**Multimedia Appendix 1.** The Color Shapes task shapes and color palette.


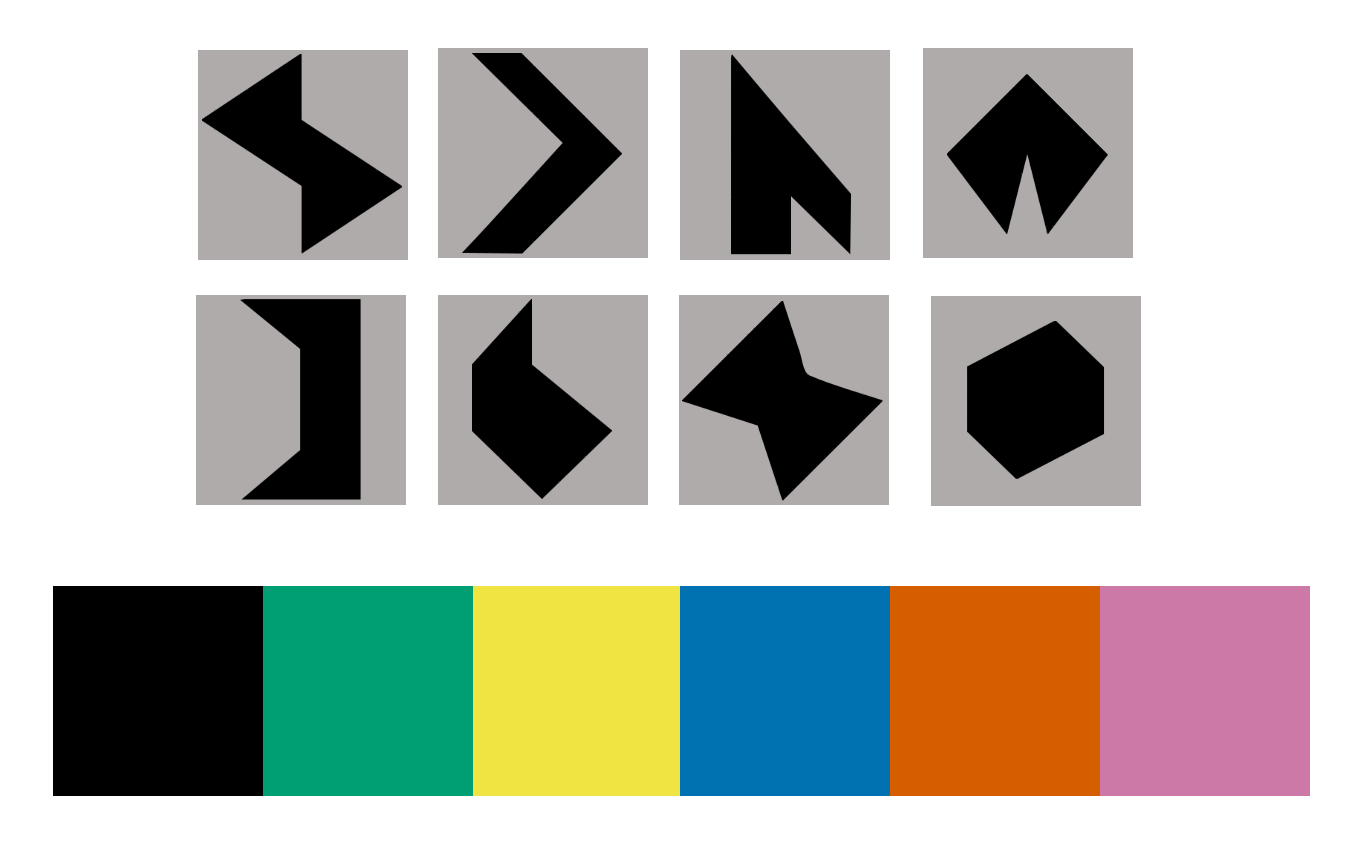


The complete set of abstract shapes (top) and color options (bottom) used to generate unique shape-color pairings in each trial of the Color Shapes task.
