# Supplementary material for: Optimizing the Color Shapes Task for Ambulatory Assessment and Drift Diffusion Modeling: A Factorial Experiment
Source: JMIR Form Res. 2025 Oct 1;9:e66300. doi: 10.2196/66300 (PMC12530164; doi:10.2196/66300)
Supplement: Multimedia Appendix 2 [file formative_v9i1e66300_app2.docx]

**Multimedia Appendix** **2.** Drift diffusion model.

### *Specification of the drift diffusion model*

To analyze response time (RT) and accuracy data from the Color Shapes task, we applied a Bayesian hierarchical drift diffusion model (DDM) [17]. The underlying assumption of the DDM is that during these tasks, participants engage in a noisy information accumulative process that stops when the cumulative evidence reaches a predetermined response boundary separation for the decision. The DDM describes such data into four parameters that capture the decision process: drift rate $\delta$, boundary separation $\alpha$, initial bias $\beta$, and non-decision time $\tau$.

More specifically, the process assumptions of the diffusion model are that a single evidence counter accumulates towards one of two decision boundaries, with a starting point that may be closer to one boundary than the other. Figure 2 illustrates the process above. Given the freedom of two decision bounds, the model accounts for two distinct types of bias in the response process. In addition to differences in the speed of evidence accumulation process (which is reflected in $\delta$), the diffusion model allows for an a-priori bias that is prior to and independent of the information accumulation process (here parameterized as a proportion, so that an initial bias $\beta$ = 0.5 implies a-priori indifference).

Overview of basic model parameters

The drift rate $\delta$ quantifies the ease of evidence processing and is generally considered not under subjective control. Higher absolute values $\left| \delta\right|$ indicate faster and accurate decisions. Conversely, lower $\left| \delta\right|$values indicate decisions slower information accumulation, thus longer RTs and with responses closer to chance. The absolute value can hence be interpreted as the ability of the person to perform the task.

The boundary separation parameter α captures the amount of information needed to execute a response. It is high for slow and deliberative responses, and low for fast and error-prone responses. This parameter can be interpreted as the speed-accuracy trade-off or the response caution of the participant during the task. It is generally considered to be under subjective control: participants can choose to prioritize speed or accuracy.

The initial bias parameter $\beta$ is the amount of information for or against either choice alternative that the participant holds before seeing a stimulus. If it is close to 1, the participant has an a-priori bias towards responding *different*; if it is close to 0, the participant has an a-priori bias towards responding *same*; if it is 0.5, the participant has no a priori bias. Participants can choose to prefer one response over another.

Finally, the non-decision time $\tau$ parameter captures all time that is taken up from the start of the task to the initiation of the information accumulation process. Commonly this includes the encoding and response processes, and it also includes other perceptual and cognitive processes depending on the individual and task.

Specification of the multilevel drift diffusion model with condition-specific means

We extended the model hierarchically to account for between- and within-subject variability in these cognitive parameters [17]. Broadly, when conducting multilevel modeling, there are two kinds of parameters. On the one hand, there are the group-level (population) parameters that capture the nomothetic patterns between groups. The individual-level parameters capture the idiographic patterns that are constrained by group-level parameters [68]. The group-level parameters can account for the extent of similarity between individuals. Group- and person-level parameters influence each other via information pooling [69].

In Equation 1 below, the drift diffusion model likelihood function is governed by the Wiener process, with parameters at the person-level and experimental condition-level. $Y_{p,c_{x},t}$ represents the observed cognitive task performance for each person, $p$, for each of the $x$ conditions ($c$), $c_{x}$ being manipulated or not (e.g., $c_{1}$ = 1 means the first condition being manipulated, as opposed to 0, $c_{1}$ = 0), on each trial, $j$. Respectively,$c_{1}$ to $c_{4}$ index the four experimental conditions: (1) study time, (2) probability of change, (3) choice urgency, and (4) probe type. The observed data include both the RT and accuracy dimensions, and follow a Wiener distribution specified by the parameters $\alpha$, $\beta$, $\tau$, and $\delta$, which were allowed to differ for each individual and across the experimental conditions. Additionally, the $\delta$, drift rates changed sign between *different* and *same* trials, captured by the $l$ index ($l$ = 1 for *different*, $l$ = 2 for *same*). Finally, person-specific $\tau_{p}$ were estimated.

$Y_{p,c_{x},t}\sim Wiener\left( \alpha_{p,c_{x}}, \beta_{p,c_{x}},\tau_{p,c_{x}},\delta_{p,c_{x},l} \right).$ (1)

Hyper-prior and prior distributions

Given that the boundary separation parameters of the DDM can only take positive values, person- and trial-specific boundary separation $\alpha$ parameters were assigned log-normal distributions as population-level distributions, specified as:

$\alpha_{p,c_{x}}\sim Lognormal(\mu_{\alpha_{c_{x}}}, \sigma_{\alpha}^{2})$,

where $\mu_{\alpha_{c_{x}}}$ is the group-level mean for the boundary separation parameter, allowed to vary between conditions $c_{x}$ and $\sigma_{\alpha}^{2}$ captures the group-level variance.

The initial bias parameter could only take values between 0 and 1, therefore we specified a normal hyperprior on its logit-transformation:

$logit(\beta_{p,c_{x}})\sim Normal(\mu_{\beta_{c_{x}}}, \sigma_{\beta}^{2})$,

where $\mu_{\beta_{c_{x}}}$ is the group-level mean for the initial bias parameter, allowed to vary between conditions $c_{x}$ and $\sigma_{\beta}^{2}$ captures the group-level variance.

For individual-level drift rate parameter estimates for the *different* trials ($l$ = 1), $\delta_{p,c_{x},l_{1}}$, the population distribution was specified as normal:

$$\delta_{p,c_{x},l_{1}}\sim Normal\left( \mu_{\delta_{c_{x}}}, \sigma_{\delta}^{2} \right),$$

where $\mu_{\delta_{c_{x}}}$ is the group-level mean for the drift rate parameter, allowed to vary between conditions $c_{x}$ and $\sigma_{\delta}^{2}$ captures the group-level variance. The drift rate for *same* trials ($l$ = 2) were parameter estimates from *different* trials that were multiplied by −1 (based on encoding RTs with *same* responses as negative) to represent processes towards the lower (*same*) decision boundary with absolute drift rate.

$\delta_{p,c_{x},l_{2}}= -1\times\delta_{p,c_{x},l_{1}}$.

For the non-decision time we assigned a uniform prior distribution, specified as a beta distribution with shape and rate parameters 1 and 1:

$\tau_{p}\sim Beta\left( 1,1 \right)$.

Finally, we specified prior distributions for the means for the three DDM parameters ($\mu_{\alpha_{c_{x}}}$, $\mu_{\beta_{c_{x}}}$, $\mu_{\delta_{c_{x}}}$) to be standard normal [Normal(0, 1)], and for the standard deviations to be uniformly distributed between 0 and 100 [Uniform(0,100)].

Bayesian implementation of the multilevel DDM

All analyses were conducted in R, Version 4.2.2. [70]. The Bayesian model was implemented in Just Another Gibbs Sampling (JAGS) [71] interfaced with R via the rjags package [72]. The Wiener distribution was specified using a custom JAGS module [73]. The analytical scripts are available on Open Science Framework [46].

Using Markov chain Monte Carlo (MCMC) algorithms implemented in JAGS. We ran 4 chains drawing 15,000 samples each with 500 burn-in samples and 500 adaptation samples per chain, resulting in 60,000 total posterior samples for each parameter.

We checked the quality of MCMC samples drawn from the posterior distribution. The effective sample size (ESS) measures and $\hat{R}$ statistics provided numerical measures of the representativeness and stability of the MCMC chains. We met general guidelines with all $\hat{R}$ values being below 1.1 [74], indicating adequate convergence within and between chains. Additionally, we had sufficient ESS (measure of independent information in autocorrelated chains) of over 1,000 [69].
