# Supplementary material for: Optimizing the Color Shapes Task for Ambulatory Assessment and Drift Diffusion Modeling: A Factorial Experiment
Source: JMIR Form Res. 2025 Oct 1;9:e66300. doi: 10.2196/66300 (PMC12530164; doi:10.2196/66300)
Supplement: Multimedia Appendix 3 [file formative_v9i1e66300_app3.docx]

**Multimedia Appendix 3.** Age distribution of participants (*N*=68).


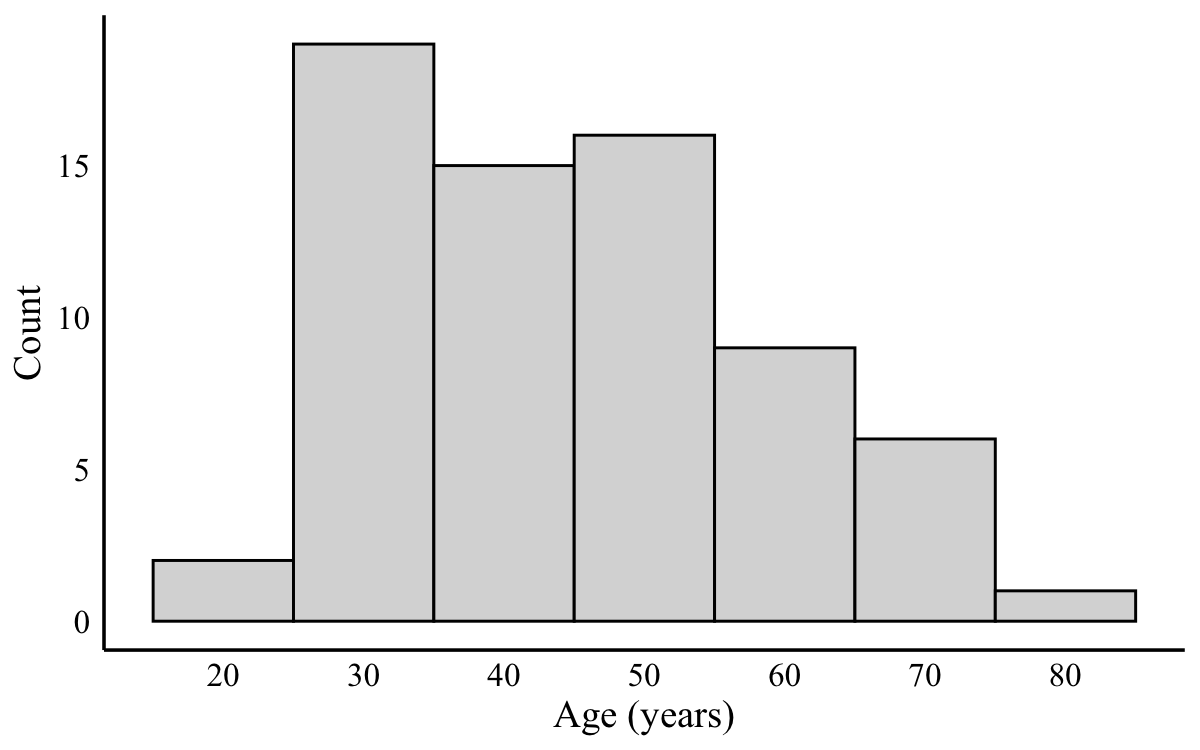


Age distribution of participant (*N* = 68), displayed in 10-year bins spanning 24 to 80 years.
