# Supplementary material for: Optimizing the Color Shapes Task for Ambulatory Assessment and Drift Diffusion Modeling: A Factorial Experiment
Source: JMIR Form Res. 2025 Oct 1;9:e66300. doi: 10.2196/66300 (PMC12530164; doi:10.2196/66300)
Supplement: Multimedia Appendix 4 [file formative_v9i1e66300_app4.docx]

**Multimedia Appendix 4.** Summary of included and excluded trials.

| Trial type | Count, n | Percent of total trials, % |
| --- | --- | --- |
| Included trials | 58,978 | 90.35 |
| Excluded trials | 784 | 1.20 |
| Invalid trials (app recording error) | 10 | 0.02 |
| RT < 200ms | 8 | 0.01 |
| RT > 7000ms | 46 | 0.07 |
| RTs timed out (choice urgency condition) | 720 | 1.10 |
| Total trials recorded | 59,762 | 91.55 |
| Total possible number of trials | 65,280 | 100 |

Descriptive summary of included and excluded trials across all participants, with a detailed breakdown of exclusion criteria and corresponding percentages based on the 65,280 total possible trials.
