# Supplementary material for: Optimizing the Color Shapes Task for Ambulatory Assessment and Drift Diffusion Modeling: A Factorial Experiment
Source: JMIR Form Res. 2025 Oct 1;9:e66300. doi: 10.2196/66300 (PMC12530164; doi:10.2196/66300)
Supplement: Multimedia Appendix 5 [file formative_v9i1e66300_app5.docx]

**Multimedia Appendix 5.**

Open-ended responses on cognitive strategy use collected during the debrief survey.

| No. | PID | Strategy reported (Yes/No) | Open-ended strategy description (if applicable) |
| --- | --- | --- | --- |
| 1 | 4000 | Yes | One-syllable nicknames for shapes with whatever color they were. Depending on the activity, I would only try to remember one or two at a time. |
| 2 | 4001 | Yes | I said what i saw aloud as quickly as i could, trying to take note of at least 2 objects and colors |
| 3 | 4002 | Yes | naming the shapes as common household items |
| 4 | 4003 | No | -- |
| 5 | 4004 | Yes | In my head I gave names to shapes. There was spool, Indiana, bridge, etc. |
| 6 | 4005 | Yes | Tried to remember two colors and corresponding shapes where possible. |
| 7 | 4006 | Yes | made up names the different shapes and said them with the color to myself; the game with the shorter time interval fornahowing the shapes I would focus on one shape to remember; in the game with the longer interval I would focus on remembering two out of the three shape/color combos |
| 8 | 4007 | Yes | I attempted to remember the color of two shapes, priortizing the shapes (box first, arrow second, etc. If a shape wasn't available then the second prioritized shape became primary for that instance.) |
| 9 | 4008 | Yes | focis on2 of 3 shaprs |
| 10 | 4009 | Yes | I recognized that you did not have to memorize all 3 colors and shapes; focusing on 2 was sufficient. Also, I identified shapes by simple names to remember them better. For example, I might remember the pattern by saying, "black fangs, pink bowtie." |
| 11 | 4010 | No | -- |
| 12 | 4011 | Yes | quiet & try to remember at least ons shape hard to explain i just ficused more |
| 13 | 4012 | Yes | look for pointy blocks and remember two colors |
| 14 | 4013 | No | -- |
| 15 | 4014 | Yes | shorthand in mind to identify the shapd |
| 16 | 4015 | Yes | I would try to memorize at least 2 shapes/colors if I could to help better recall. |
| 17 | 4016 | Yes | tried memorizing 2 shapes and colors |
| 18 | 4017 | Yes | association- but had trouble concentrating - easily distracted |
| 19 | 4018 | No | -- |
| 20 | 4019 | No | -- |
| 21 | 4020 | Yes | tried to remembrr 2 colord |
| 22 | 4021 | Yes | i tried to only concentrate on two colors /shapes and i said them out loud if the timer was slower |
| 23 | 4022 | Yes | I gave the shapes names like space ship, arrow, 7, house, and said the colors for at least 2 of them |
| 24 | 4023 | Yes | tried not to be stressed out |
| 25 | 4024 | Yes | created one word descriptors for each shape and memorized the color and nickname for two of the three objects before the first screen disappeared. |
| 26 | 4025 | Yes | focused on 1 or 2 figures instead of all 3 |
| 27 | 4026 | Yes | color patterns stayed the same and only had to remember one for some games |
| 28 | 4027 | No | -- |
| 29 | 4028 | N/A | N/A |
| 30 | 4029 | Yes | remembering objects from largest to smallest with their corresponding colors |
| 31 | 4030 | Yes | pick two out on the to remember, pick triangle shapes as easiest to remember |
| 32 | 4031 | N/A | N/A |
| 33 | 4032 | Yes | Tried to focus on just two figures when it was moving quickly, also tried to focus on rectangles and greater than symbol since they were uique and not similar to other figures. |
| 34 | 4033 | No | -- |
| 35 | 4034 | Yes | focused on esdy to find shapes |
| 36 | 4035 | Yes | said either aloud or in my head the color and the shape of one or 2 objects. then on the next screen i'd be able to on theory know whether they were correct. since some of the objects were "irregular" i made up a shape name for them. one was zig zag, another was parentheses |
| 37 | 4036 | Yes | focused on color and shape of 2 of the 3 especially on the ones that were presented very quickly. also i said the names of thr colors out loud |
| 38 | 4037 | Yes | concentrating on shapies that were not similar to any of the others, trying to remember 2/3 shapes |
| 39 | 4038 | Yes | tried to verbalize shapes to remember a bit better |
| 40 | 4040 | Yes | remembering one shape and its color |
| 41 | 4041 | No | -- |
| 42 | 4042 | Yes | I tried to focus on the larger shapes and their colors . |
| 43 | 4043 | Yes | watching only one or two shapes or colors |
| 44 | 4044 | Yes | identify the one or two images with the sharpest angles work to remeber the sharpest angle color and rhr next sharpest angle color, in the game with all 3 shapes reapearjng, focus only 1 shapes - with the sharpest angle |
| 45 | 4045 | Yes | I began to try to memorize two close-together shapes/colors as they flashed on the screen, then get a glimpse of the third before they all disappeared. |
| 46 | 4046 | Yes | associating each shape with a name such as pac for the pac man shape. Also tried to learn to quickly pan the shapes and not fixate on each one as i tried to memorize what shape and color each one was. |
| 47 | 4047 | Yes | naming shapes and colors |
| 48 | 4048 | Yes | focused on one object and peripheral colors on others: easier to subconsciously see. sometimes i meditated first those were better days |
| 49 | 4049 | Yes | I tried to remember shapes in the same order each time and said the color names in my head. |
| 50 | 4050 | Yes | began to focus on 1 or two shapes with their color. this depended on there proximity to each other. easier when grouped. I would estimate this worked 50 % of the time while remainder was a gut feeling. the first three days I tried to take a mental picture of the shapes the answered based in feeling. |
| 51 | 4051 | Yes | noticd two shapes instead of all three, thats enough info to know if theres a difference |
| 52 | 4052 | Yes | trying to name the shapes and colors (black tall, pink arrow, blue z, orange hourglass, etc) |
| 53 | 4053 | Yes | I tried focusing very hard on two out of the three shapes and quickly thinking about the third |
| 54 | 4054 | Yes | Tried to focus on very familar objects first. like the arrow or the damond. |
| 55 | 4055 | Yes | saying the colors outloud to see if I could remember them better. |
| 56 | 4056 | Yes | i ranked the shapes and used am arbitrary order to remember shapes and colors. due to the speed of the game, sometime, i risk matching only 2 shapes and colors and guess the last using visual memory. i tried to vocalize the shapes one time, but it did not help much. |
| 57 | 4057 | N/A | N/A |
| 58 | 4058 | Yes | try to remember only one or two shapes and colors, not all three. |
| 59 | 4059 | No | -- |
| 60 | 4060 | Yes | I tried to focus on just two of the objects per (first) screen, allowing the third item to be a default when viewing the second (comparison) screen. |
| 61 | 4061 | Yes | Tried to say aloud at least 2 of the colors/shapes |
| 62 | 4062 | N/A | N/A |
| 63 | 4063 | Yes | tried to memorize shapes first then colors or memorize two shapes color |
| 64 | 4064 | Yes | I would focus on two shapes and their colors, to attempt to better guess what popped up on the screen next |
| 65 | 4066 | Yes | i would focus on one shape and its color |
| 66 | 4067 | Yes | remembering the shapes as a whole on the screen instead of remembering them individually |
| 67 | 4068 | Yes | my strategy was to name certain shapes amdntheir colors outloud plus giving some shapes names like Couch , Stop Sign , Plane , Drum , etc |
| 68 | 4070 | Yes | On the very quick exercises, I made sure to focus on two objects if not all three. On the third object, I at least wanted to remember the color to help my decision. And if I had no idea, I hit "different." |
